# Oral Delivery of Liraglutide-Loaded Zein/Eudragit-Chitosan Nanoparticles Provides Pharmacokinetic and Glycemic Outcomes Comparable to Its Subcutaneous Injection in Rats

**DOI:** 10.3390/pharmaceutics16050634

**Published:** 2024-05-09

**Authors:** Jeferson Ziebarth, Letícia Marina da Silva, Ariane Krause Padilha Lorenzett, Ingrid Delbone Figueiredo, Paulo Fernando Carlstrom, Felipe Nunes Cardoso, André Luiz Ferreira de Freitas, Amanda Martins Baviera, Rubiana Mara Mainardes

**Affiliations:** 1 Laboratory of Nanostructured Formulations, Universidade Estadual do Centro-Oeste, Alameda Élio Antonio Dalla Vecchia St., 838, Guarapuava 85040-167, PR, Brazil; jeferson_ziebarth@outlook.com (J.Z.); leticiamarina.ds@gmail.com (L.M.d.S.); arianekrause@hotmail.com (A.K.P.L.); 2 Department of Clinical Analysis, School of Pharmaceutical Sciences, São Paulo State University, Rodovia Araraquara Jaú, Km 1–s/n, Araraquara 14800-903, SP, Brazil; delboneingrid@gmail.com (I.D.F.); fernando.carlstrom@unesp.br (P.F.C.); felipe.n.cardoso@unesp.br (F.N.C.); andre.f.freitas@unesp.br (A.L.F.d.F.); amanda.baviera@unesp.br (A.M.B.); 3 Department of Pharmacy, Universidade Estadual do Centro-Oeste, Alameda Élio Antonio Dalla Vecchia St., 838, Guarapuava 85040-167, PR, Brazil

**Keywords:** type 2 diabetes mellitus, glucagon-like peptide-1, zein nanoparticles, oral bioavailability

## Abstract

Liraglutide (LIRA) is a glucagon-like peptide-1 (GLP-1) receptor agonist renowned for its efficacy in treating type 2 diabetes mellitus (T2DM) and is typically administered via subcutaneous injections. Oral delivery, although more desirable for being painless and potentially enhancing patient adherence, is challenged by the peptide’s low bioavailability and vulnerability to digestive enzymes. This study aimed to develop LIRA-containing zein-based nanoparticles stabilized with eudragit RS100 and chitosan for oral use (Z-ERS-CS/LIRA). These nanoparticles demonstrated a spherical shape, with a mean diameter of 238.6 nm, a polydispersity index of 0.099, a zeta potential of +40.9 mV, and an encapsulation efficiency of 41%. In vitro release studies indicated a prolonged release, with up to 61% of LIRA released over 24 h. Notably, the nanoparticles showed considerable resistance and stability in simulated gastric and intestinal fluids, suggesting protection from pH and enzymatic degradation. Pharmacokinetic analysis revealed that orally administered Z-ERS-CS/LIRA paralleled the pharmacokinetic profile seen with subcutaneously delivered LIRA. Furthermore, in vivo tests on a diabetic rat model showed that Z-ERS-CS/LIRA significantly controlled glucose levels, comparable to the results observed with free LIRA. The findings underscore Z-ERS-CS/LIRA nanoparticles as a promising approach for oral LIRA delivery in T2DM management.

## 1. Introduction

It is estimated that the global incidence of diabetes mellitus (DM) in 2021 (latest global survey) will be 537 million people, representing 9.3% of adults aged between 20 and 79 years [1]. Projections indicate that this number is expected to increase to 783 million by 2045 [1,2]. It is worth noting that more than 90% of cases of diabetes mellitus correspond to type 2 [3,4,5]. Type 2 diabetes (T2D) represents a chronic metabolic condition characterized by insulin resistance and insufficient insulin production by pancreatic cells, associated with lifestyle factors such as inadequate diet and lack of physical activity [6].

The treatment of type 2 diabetes is approached comprehensively, incorporating strategies that aim to control blood glucose, improve insulin sensitivity and manage related risk factors [2]. A crucial initial intervention includes adopting a healthy lifestyle characterized by a balanced diet rich in complex carbohydrates, fiber, lean proteins and healthy fats, combined with regular physical activity [7].

In the context of the treatment of T2D, liraglutide (LIRA), a glucagon-like peptide-1 (GLP-1) receptor agonist, stands out as an effective option [8]. In addition to stimulating the release of insulin in response to high glucose, LIRA delays gastric emptying, promoting satiety and aiding weight loss [9]. These benefits contribute significantly to glycemic control and improved quality of life for patients [8,10]. The pharmacokinetic characteristics of LIRA allow for an extension of exposure for 24 h, meeting the need for glycemic control throughout the day with once-daily administration [10].

However, the use of LIRA faces challenges, especially with regard to adverse effects, such as nausea and diarrhea, in addition to causing liver and pancreatic problems [10,11,12]. There is also a need for administration by daily subcutaneous injection, which may impact treatment adherence [13]. Furthermore, oral forms would provide better adherence to treatment, however, as it is a protein, administration via this route is impossible due to the degradation of the molecule in gastric juice [10]

Pharmaceutical nanotechnology is an approach to improve the oral administration of molecules composed of proteins or peptides, overcoming absorption and gastrointestinal stability challenges [14,15]. The use of nanocarriers, such as nanoparticles (Nps) and liposomes, seeks to improve the bioavailability of these molecules, in addition to assisting in the pharmacokinetic and dynamic properties of the molecules, although it faces obstacles such as the mucosal barrier, enzymatic degradation and irregular absorption [16].

Within this context, zein emerges as a promising candidate in pharmaceutical nanotechnology. Zein, a protein derived from corn classified as prolamin, has distinct physicochemical characteristics with notable insolubility in water. However, its high solubility in organic solvents, such as ethanol, methanol and acetone, enables the formation of films and coatings from zein solutions [17]. This property has significant applications in coatings for pharmaceutical tablets, capsules and the formation of nanostructures for drug loading and targeting [18].

Zein demonstrates favorable compatibility with a variety of polymers, giving it versatility in blended formulations to meet specific requirements [19]. Additionally, its biodegradability, combined with renewable origin, contributes to its appeal in applications aimed at sustainable practices in the pharmaceutical industry and other areas [20]. Furthermore, zein exhibits satisfactory thermal stability at moderate temperatures, favoring formulation processes that involve controlled heating [20,21].

The use of zein in the formation of Nps serves as efficient vehicles for the controlled delivery of drugs, improving the solubility, bioavailability and stability of drugs, peptides or proteins in the gastrointestinal tract [20,22,23]. Through their solubility, the formation of nanoparticles occurs through self-assembly, promoting the encapsulation of various compounds, where the average diameter varies between 100 and 300 nm [22].

Eudragit RS100 (ERS100), a pH-sensitive synthetic polymer, is increasingly recognized for its application in drug formulation [24,25]. ERS100 is a favorable choice for producing zein nanoparticles owing to its notable resistance within the gastrointestinal tract and its efficacy in targeted drug delivery [25]. Additionally, chitosan (CS), a naturally occurring biodegradable polymer with low toxicity, is extensively utilized in nanotechnology, particularly for the encapsulation of biomolecules for oral administration, and its mucoadhesive properties and robust stability in the gastrointestinal environment make it an excellent stabilizer for zein Nps [26].

Within the scope of this work, we aimed to develop zein Nps for the encapsulation of LIRA, utilizing ERS100 and CS as stabilizers targeted for oral delivery. The performance of these Nps was assessed by analyzing their pharmacokinetic properties following oral administration in healthy rats, as well as their ability to regulate blood glucose levels in a T2D rat model.

## 2. Materials and Methods

### 2.1. Materials

Liraglutide (injection, 3.0 mg) was purchased from Saxenda© (Novo Nordisk, São Paulo, Brazil). Zein, low molecular mass chitosan (50–190 kDa, 75–85% deacetylated), streptozotocin and formic acid were purchased from Sigma-Aldrich (St. Louis, MO, USA). Eudragit© RS100 was obtained from Evonik (Berlin, Germany). Absolute ethanol was purchased from Synth^®^ (São Paulo, Brazil). HPLC-grade acetic acid was obtained from Vetec Química Fina (Duque de Caxias, Brazil). Purified water was obtained using a Milli-Q Plus system (Millipore, Burlington, MA, USA) with a conductivity of 18 MΩ.

### 2.2. Development of Zein/Eudragit-Chitosan Nanoparticles Containing Liraglutide

Zein/eudragit-chitosan nanoparticles containing liraglutide (Z-ERS-CS/LIRA) were developed using the anti-solvent precipitation technique [27,28]. Zein and Eudragit RS100 (ERS100) were dissolved in an ethanol/water solution (87:13, *v*/*v*), at a concentration of 10.0 mg/mL and 9.0 mg/mL, respectively, under magnetic stirring at 600 rpm, for 1 h 30 min. Chitosan (CS) was dissolved in a 1% (*v*/*v*) acetic acid solution at a concentration of 0.25 mg/mL, under magnetic stirring at 500 rpm for 24 h. Following the dissolution period, the CS solution was vacuum-filtered, and the pH was adjusted to 4.0 using a 1.0 mol/L HCl solution, then stored under refrigeration. In a Falcon-type tube, 1.5 mL of zein and 1.2 mg of liraglutide (LIRA) were combined, and this mixture was incubated with orbital rotation at 150 rpm and 25 °C for 2 h. Subsequently, 1.5 mL of the ERS100 ethanol solution was added to the tube for an additional hour of incubation. After incubation, the solution containing zein, LIRA, and ERS100 was transferred to a beaker with 6 mL of CS solution and stirred magnetically at 1500 rpm for 15 min. For complete ethanol evaporation, the Nps suspension was taken to a rotary evaporator set at 45 °C. Post-evaporation, the samples were ultracentrifuged at 26,500× *g* for 20 min at 20 °C. The supernatant was set aside for further analysis, and the precipitate was re-suspended in water. The final pH value of the nanoparticle suspension was 4.5.

### 2.3. Physicochemical Characterization

#### 2.3.1. Mean Size, Polydispersity Index and Zeta Potential Analysis

The mean size and polydispersity index (PDI) of the Nps were determined using dynamic light scattering (DLS) via the Brookhaven 90 Plus instrument (Brookhaven Instruments Corp., Holtsville, NY, USA). The samples were diluted in ultrapure water (1:100) and placed in a cuvette with a lid. Zeta potential was determined from the electrophoretic mobility of the suspended Nps. The measurements were carried out using the ZetaSizer equipment (ZS-Malvern^®^, Malvern, UK). The samples were diluted (1:100) in a 1 mM KCl aqueous solution and placed in the electrophoretic cell, where a potential of ±150 mV was established. Throughout both tests, the pH of the nanoparticle solutions was consistently maintained at approximately 4.5. This was conducted to ensure the pH closely matched the final pH of the Nps suspension obtained, which was also approximately 4.5, thus ensuring consistent conditions for analysis.

#### 2.3.2. Morphological Analysis

Morphological analysis of Z-ERS-CS/LIRA was evaluated in a scanning electron microscopy (SEM) employing the MIRA3 LM instrument from Tescan Orsay Holding. SEM images were acquired at magnifications of 9000 and 21,000 times. For this analysis, a drop of Nps dispersions was distributed on a metal support (stubs), and after drying the samples at room temperature, the stubs were metallized with gold under vacuum to be analyzed.

#### 2.3.3. Determination of the Encapsulation Efficiency

Quantification of the LIRA encapsulated within Nps was performed by indirect analysis using High-Performance Liquid Chromatograph (HPLC) methodology. Analysis was performed using the HPLC Waters^®^ 2695 Alliance with a Photodiode Array (PDA) detector 2998. Chromatographic conditions consisted of ultrapure water acidified with 0.5% acetic acid and acetonitrile (55:45, *v*/*v*) as the mobile phase, with a C18 column (Atlantis T3 Waters^®^, 250 mm × 4.6 mm, 5 μm), injection flow rate of 1.0 mL/min, injection volume of 20 μL, column temperature 30 °C, detection wavelength at 271 nm and run time of 6 min.

The analyte consisted of the supernatant obtained following the ultracentrifugation step of the Nps. Encapsulation Efficiency (EE) was calculated based on the difference between the initial amount of LIRA added during the Nps preparation and the amount of LIRA found free in the supernatant, not incorporated into the Nps. The encapsulation efficiency was determined using Equation (1):

EE (%) = ((LIRAi − LIRAf)/LIRAi) × 100(1)

where LIRAi is the initial amount of LIRA added to the formulation and LIRAf is the amount of LIRA not incorporated into the Nps, as quantified by the analytical method.

EE values are reported as mean ± standard deviation.

### 2.4. In Vitro Release Profile Assay

In vitro release assay was performed using a Franz-type vertical diffusion cell system (Hanson^®^). A phosphate buffer solution (PBS, 50 mM, pH = 7.4) was used as a release medium, which was added to the cells, with controlled temperature and agitation (37 °C and 300 rpm). Z-ERS-CS/LIRA formulations containing approximately 90 µg of LIRA were deposited onto 0.45 μm nitrocellulose membranes and at predetermined intervals (0.5, 1, 2, 4, 8, 12 and 24 h) an aliquot of the release medium (1 mL) was collected and replaced with the same volume of fresh medium. Collected samples were filtered through a 0.22 μm membrane for subsequent HPLC analysis. For the determination of the LIRA release mechanism from Nps, the data obtained from the release were analyzed by the software KinetDS^®^ (version 3.0) from different mathematical models: zero-order, first-order, second-order and third-order models, Higuchi model, Weibull model and Hixson–Crowell. The release exponent “n” was calculated according to the Korsmeyer-Peppas model.

Moreover, the in vitro release of LIRA was evaluated in simulated gastric fluid (SGF) and simulated intestinal fluid (SIF) using the dialysis membrane technique. SGF is composed of KCl 50 mM and pepsin 1% with the pH adjusted to 1.2 using 2.0 mol/L HCL, while SIF consists of KH_2_PO_4_ 50 mM, NaOH 15 mM, pancreatin 1%, with the pH adjusted to 6.8 with 2.0 mol/L HCl [29]. In this experiment, an aliquot of 500 μL of Z-ERS-CS/LIRA Nps was placed inside a dialysis bag (MWCO 14,000). The bag was immersed in reservoirs containing 15 mL of SGF pH 1.2 supplemented with pepsin for a period of 2 h. Subsequently, the dialysis bag was immersed in SIF with pH 6.8 containing pancreatin and incubated for another 4 h. Release media were maintained at 37 ± 0.5 °C with magnetic stirring at 150 rpm. At specific time points (0.25, 0.5, 1, 2, 3, 4, 5, and 6 h), aliquots were collected, and the withdrawn volume was replenished with fresh SGF or SIF. The collected samples were filtered through 0.22 μm PVDF membranes and subjected to HPLC analysis.

### 2.5. Pharmacokinetic Study

#### 2.5.1. Ultra-Performance Liquid Chromatography Analysis

Ultra-performance liquid chromatography (Acquity UPLC© Waters, Milford, MA, USA), coupled with a triple quadrupole mass spectrometer (XEVO-TQD, Waters^®^) equipped with a Z sprayTM electrospray ionisation source (Waters^®^, Milford, MA, USA), was employed to detect, and quantify LIRA levels in rat plasma after treatment. An ACQUITY UPLC BEH C18 reversed-phase chromatographic column (50 mm × 2.1 mm; 1.7 µm) was used, with a mobile phase composed of acetonitrile and ultrapure water, both acidified with 0.1% formic acid, eluted in gradient mode, with varying proportions (20/80, 45/55, 60/40, 80/20, 90/10, 80/20, 30/70, 20/80) at specific time intervals (0, 1.5, 2.0, 2.5, 3.0, 3.5, 4.5, 6.0 min), with a flow rate of 0.3 mL/min, injection volume of 2 µL, with a total analysis time of 7.5 min. The samples were maintained at a temperature of 10 °C in the sampler, while the column oven was maintained at 40 °C. Detection by mass spectrometry was carried out in positive electrospray ionisation (ESI+) mode, in multiple reaction monitoring (MRM) mode, with a source temperature of 150 °C, capillary voltage equal to 3.5 kV, desolvation temperature of 600 °C, desolvation flow of 1000 L/h and gas flow in the cone of 50 L/h. The retention times for internal standard (IS), insulin detemir, and LIRA were determined as 3.15 min and 3.39 min, respectively. The transitions set at *m*/*z* 938.7 → 1064.1 for LIRA and *m*/*z* 1184.7 → 454.4 for insulin detemir [30].

#### 2.5.2. Pharmacokinetic Assay

Firstly, for the pharmacokinetic test, approval was required from the Ethics Committee on the Use of Animals of the Universidade Estadual do Centro-Oeste, obtained through protocol number 009/2022. Adult male Wistar rats weighing between 160 and 220 g were utilized for the study. The animals were kept in polyethene boxes containing wood shavings and placed in a chamber with a 12-h light/dark cycle, 35% humidity, 22 °C, with water and food ad libitum for up to 10 h before and 2 h after drug administration. The rats were separated into two groups: Group A (*n* = 6), which received 200 µg/kg of LIRA subcutaneously, and Group B (*n* = 6), which received Nps containing around 200 µg/kg of LIRA, orally, through gavage. Blood samples were collected from the rats at predetermined time intervals (0.5, 1, 2, 4, 8, 12, and 24 h) following administration. Subsequently, the samples (200 µL) were transferred to microtubes containing heparin.

#### 2.5.3. Sample Preparation

Blood samples were collected via the tail vein and transferred to heparinized microtubes. Centrifugation at 1370× *g*, 25 °C for 10 min was conducted to extract plasma. Thereafter, liquid-liquid extraction with acetonitrile was performed. Specifically, a 50 µL plasma sample aliquot was combined with 250 μL of acetonitrile containing the IS with a concentration fixed at 100 ng/mL. After centrifugation at 1370× *g* for 10 min at 25 °C, the supernatant was filtered using 0.22 µm syringe filters (Filtrilo, PVDF, Colombo, Brazil), and transferred to an injection vial for subsequent analysis using UPLC-MS/MS [30].

#### 2.5.4. Data Analysis

Pharmacokinetic parameters were estimated using the PKSolver plugin for Microsoft Excel. Non-behavioral pharmacokinetic analysis was used to determine peak plasma concentration (C_max_), time at which peak plasma concentration occurred (T_max_), area under the curve of plasma drug concentration versus time (AUC_0–24h_), elimination half-life (T_1/2_), elimination constant (K_el_) and clearance (Cl).

### 2.6. In Vivo Type 2 Diabetes Mellitus Experimental Model

For this test, 50 male Wistar rats were used, with an average weight of 180 ± 10 g, purchased from the company Animais de Laboratório Criação e Comércio LTDA-ANILAB (Paulínia, SP, Brazil). The animals were kept in polyethene boxes, under a 12-h light/dark cycle, at a temperature of 22 ± 2 °C, receiving food and water ad libitum. The experimental procedures were approved by the Ethics Committee on the Use of Animals of the Faculty of Pharmaceutical Sciences at UNESP (CEUA/FCF/CAr n° 10/2022).

Experimental type 2 diabetes mellitus (T2D) was induced from a single dose of 45 mg of streptozotocin (STZ)/kg of animal body weight, solubilized in citrate buffer (0.01 mol/L, pH = 4.5), via intraperitoneal (i.p.) route. After four days of STZ administration, body weight and blood glucose were analyzed, where animals that presented blood glucose levels > 250 mg/dL were considered diabetic. Finally, using blood glucose and body weight values, the animals were paired and distributed into different experimental groups, namely: Group ND consisted of non-diabetic animals that did not receive STZ, with a sample size of *n* = 10; Group DNT included diabetic animals that went without treatment, also *n* = 10; Group DNB encompassed diabetic animals that were administered blank Nps without any drug, given orally twice daily, *n* = 10; Group DNL was made up of diabetic animals that received Nps containing approximately 72 µg of LIRA, administered orally twice a day, *n* = 10; and finally, Group DLS included diabetic animals that were treated with roughly 72 µg of LIRA via subcutaneous injection, twice a day, with a sample size of *n* = 10.

The study was carried out for 28 days, with body weight being checked on days 0, 7, 14, 21 and 28, while blood glucose was analyzed on days 0, 10, 20 and 28. Blood samples (100 µL) for measurement of blood glucose levels were collected through the animals’ tail vein, placed in microtubes containing heparin, subjected to centrifugation at 700× *g* for 10 min and subsequently separated from the plasma.

### 2.7. Statistical Analysis

All experimental data were presented as mean values ± standard deviation. For the physicochemical parameters and pharmacokinetic studies, *n* was 6. For in vitro release assay *n* = 3, and for the in vivo diabetes assays, *n* equaled 10. Statistical analyses were performed using ANOVA with a confidence level of 95%, followed by Tukey’s post-test. (GraphPad Prism V.10, Free Trial).

## 3. Results and Discussion

### 3.1. Development of Zein/Eudragit-Chitosan Nanoparticles Containing Liraglutide

Z-ERS-CS/LIRA Nps were obtained using the anti-solvent precipitation technique, also recognized as the anti-solvent nanoprecipitation method. This approach involves the incorporation of an anti-solvent, typically water, which may or may not contain surfactants, into a hydroalcoholic zein solution. The concentration of ethanol in this solution generally ranges from 60% to 90% [31]. Zein-derived Nps are particularly adept at encapsulating hydrophobic compounds. This encapsulation efficiency is due to the interaction between the hydrophobic compounds and the non-polar regions of zein. The process is facilitated by the increased polarity upon the addition of water, which causes the hydrophilic ends of zein to orient towards the aqueous environment and the hydrophobic regions to align towards the interior of the Nps. This orientation forms a protective niche for the hydrophobic compounds [32,33]. Given the pronounced hydrophilic nature of LIRA, its interaction with zein is impaired during Nps formation. To enhance this interaction and stabilize the resultant Nps, ERS 100 was introduced into the zein/LIRA blend. The inclusion of ERS 100 fosters a more synergistic interaction between LIRA and zein. Concurrently, it aids in stabilizing the Nps by facilitating their interaction with CS, which is present in the aqueous phase. CS, under acidic conditions, adopts a positive charge and, upon Nps formation, adorns their surfaces, imparting a positive charge.

### 3.2. Physicochemical Characterization

The results of the main physicochemical characterization are presented in Table 1. Blank Z-ERS-CS Nps (without LIRA) yielded an average diameter of 225.9 nm and 238.6 nm for those incorporating LIRA (Z-ERS-CS/LIRA Nps), demonstrating negligible variation in size. The PDI of Z-ERS-CS Nps was 0.070, while the Z-ERS-CS/LIRA Nps presented a slightly elevated PDI of 0.099, indicative of high homogeneity in the Nps size in both formulations. Zeta potential measurements for both Nps formulations were approximately +40 mV, attributable to the presence of CS on the nanoparticles’ surface, which imparts a positive charge. The Z-ERS-CS/LIRA Nps exhibited an average encapsulation efficiency of 41.1%, aligning with findings in comparable studies. Bao et al. (2023) reported similar diameter ranges for their zein/cholic acid/rhamnolipid-based Nps designed for LIRA encapsulation, and Ji et al. (2018) observed comparable size distributions and PDI values in their development of zein/carboxymethylated short-chain amylose complex for insulin delivery [34,35]. The pronounced positive zeta potential aligns with studies by Pauluk et al. (2019) and Zhou et al. (2021), who also employed CS to coat zein Nps, achieving zeta potential values of +30 mV and +58.5 mV, respectively [36,37].

It is noteworthy that the encapsulation efficiency (EE) of hydrophilic compounds generally trends lower than that of hydrophobic ones, given the tendency of hydrophilic molecules to partition into the aqueous phase during Nps formation [38,39]. Nevertheless, the 41% EE achieved for LIRA in our study is consistent with the work of Donsì and co-authors (2017) for the encapsulation of the hydrophilic agent epigallocatechin gallate in zein nanoparticles (EE = 36–46%), and by Bao et al. (2020) for the encapsulation of the GLP-1 analogue exenatide (EE = 31–54%), further validating our methodology [40,41].

**Figure 1:**
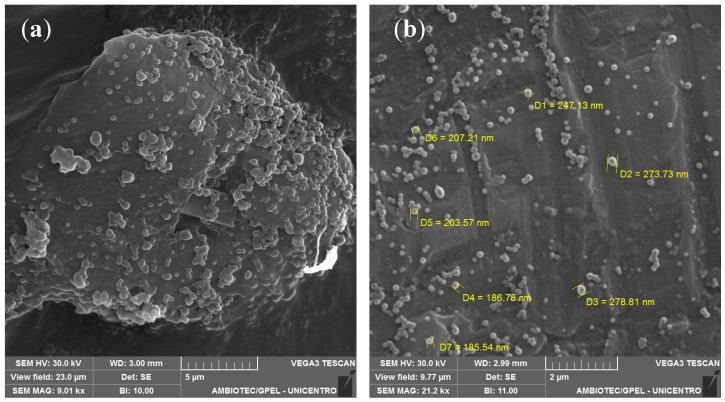
Scanning electron microscopy (SEM) images of liraglutide-containing zein/eudragit-chitosan nanoparticles (Z-ERS-CS/LIRA): (**a**) Magnification of 9.01 k; (**b**) Magnification of 21.2 k.

SEM imaging of the Z-ERS-CS/LIRA Nps, as displayed in Figure 1, offers a detailed visualization at varying magnifications. At 9.0 k-fold magnification, as seen in Figure 1a, there is an observable formation of particle aggregates. Such aggregation is likely attributable to the intermolecular forces between CS chains, a phenomenon that is exacerbated by the dehydration inherent in the sample drying phase, corroborating observations from similar studies where CS is a component of the Nps corona [36,42,43]. At an enhanced magnification of 21.2 k, Figure 1b permits a more refined examination of the Nps. Despite the presence of some aggregation, the Nps predominantly exhibit a spherical and smooth topology.

### 3.3. In Vitro Release Profile Assay

**Figure 2:**
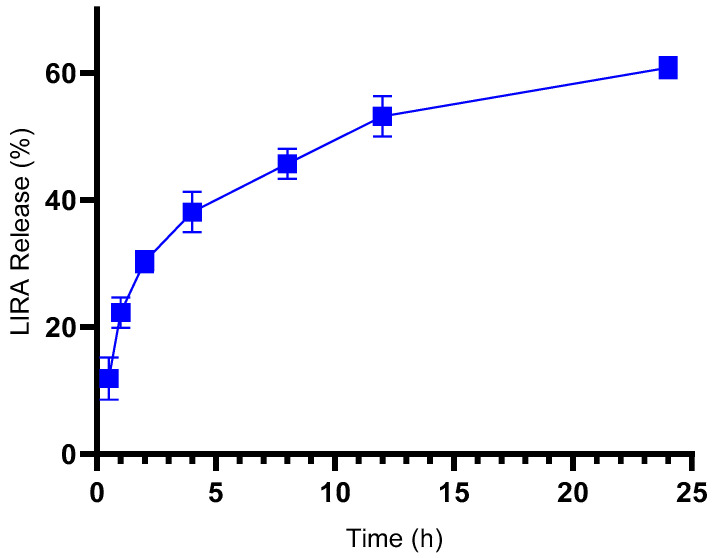
In vitro release profile of liraglutide from zein/eudragit-chitosan nanoparticles (Z-ERS-CS/LIRA) in PBS solution (50 mM) at 37 °C over a 24 h period (*n* = 3).

The in vitro release profile of LIRA from the Z-ERS-CS/LIRA Nps, as illustrated in Figure 2, follows a biphasic pattern where the release of LIRA can be modulated by gradient concentration. There is an initial rapid release of approximately 38% of LIRA from the Nps within the first hour, which is then followed by a steady and controlled release, eventually resulting in approximately 61% of LIRA being released at the 24-h mark. Comparable release dynamics were documented by Vale et al. (2024), who reported the dispersion of the hydrophilic agent Epigallocatechin-3-gallate from zein Nps, with a release reaching nearly 70% within a day [44]. Bao et al. (2023) observed a release range of 70–80% for LIRA from zein Nps over the same duration, applying stabilizing agents. Although slight variances were noted across different formulations, these did not significantly impact the release from the zein matrix [34].

Subsequent analysis applied release kinetics to pinpoint the mechanism of LIRA liberation from the Nps. The correlation coefficient (r) was utilized as a criterion for discerning the most suitable mathematical model that aligns with the drug release pattern (Table 2). The Weibull model, presenting an r-value of 0.961, emerged as the most congruent with the release date. This model is extensively used to characterize drug release, encompassing various processes such as diffusion, dissolution, or their confluence. Another model, the Korsmeyer-Peppas, calculates the exponent ‘n’ to elucidate the release mechanism further. An ‘n’ value ≤ 0.45 denotes a Fickian diffusion, primarily driven by the drug’s permeation through the matrix’s structure. Conversely, ‘n’ values ≥ 0.85 suggest a non-Fickian release, indicative of matrix erosion. Values between 0.45 < ‘n’ < 0.85 represent an amalgamation of diffusion and erosion, termed an anomalous process [45,46,47]. The Korsmeyer-Peppas model yielded an ‘n’ value of 0.394, inferring that the LIRA dissemination from the Nps ensued through a Fickian diffusion process.

The in vitro release data suggest a nuanced encapsulation behaviour of LIRA within the Nps matrix. While the slight burst effect observed may indicate that a portion of the drug is accessible on the surface of the nanoparticles, the dominant Fickian diffusion release kinetics strongly suggests that a significant amount of LIRA is encapsulated within. The fact that only 60% of the drug was released over 24 h further supports the idea that a substantial proportion of LIRA is deeply embedded in the Nps structure and not merely adsorbed on the surface. This encapsulation not only potentially enhances the stability of LIRA within the system but also suggests a controlled release mechanism that could be beneficial for sustained therapeutic effects.

**Figure 3:**
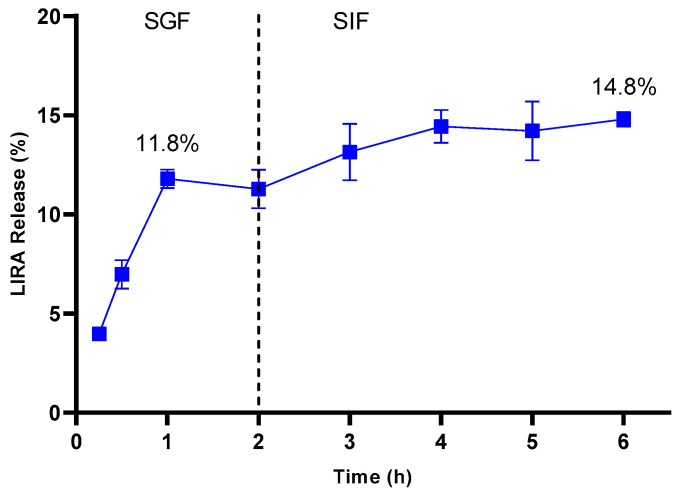
In vitro release profile of liraglutide (LIRA) from zein/eudragit-chitosan nanoparticles containing LIRA (Z-ERS-CS/LIRA) in simulated gastric (SGF—pH 1.2) and intestinal fluids (SIF—pH 6.8) (*n* = 3).

The evaluation of the release profile in simulated gastrointestinal fluid was conducted to ascertain the potential for oral delivery of LIRA from Z-ERS-CS/LIRA Nps. The investigation involved a sequential exposure of the Nps to SGF with a pH of 1.2 for two hours, succeeded by a medium transition to SIF with a pH of 6.8 for an additional four hours. Release results from three independent samples are shown in Figure 3. Notably, the Nps demonstrated minimal release of LIRA in both SGF and SIF, culminating in a peak release of 11.8% and 14.8%, respectively, at the six-hour. This release pattern is indicative of the Nps’ robustness and stability, which preserve their structural integrity even with fluctuating pH levels and the enzymatic activity characteristic of the digestive process.

Comparatively, the study performed by Bao et al. (2023) reported a release rate of approximately 18% for LIRA from zein-based Nps in SGF within a two-hour window. This finding underscores the efficacy of zein Nps, integrated with various stabilizing agents, in safeguarding LIRA against the harsh conditions of an acidic milieu [34].

### 3.4. Pharmacokinetic Study

**Figure 4:**
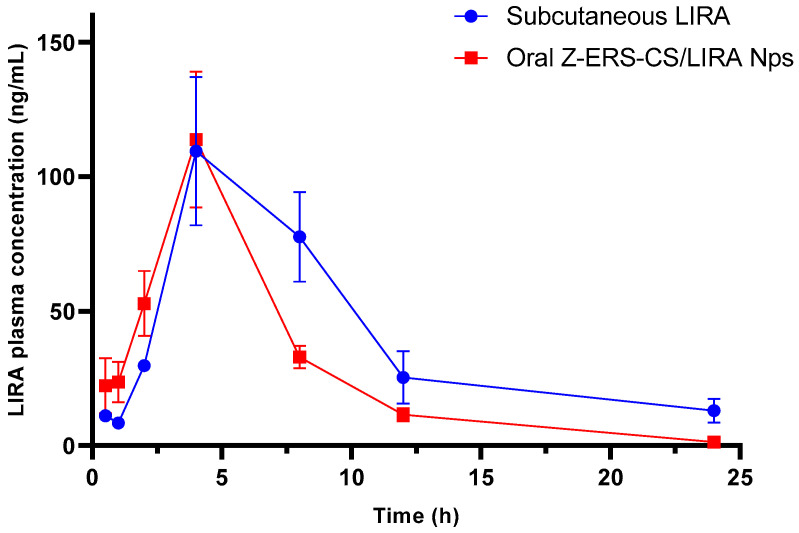
Comparative plasma concentration-time profiles of liraglutide (LIRA) in rats (*n* = 6): subcutaneous injection of free LIRA vs. oral delivery of LIRA-containing zein/eudragit-chitosan nanoparticles (Z-ERS-CS/LIRA). The administered dose of LIRA was 200 µg/kg for both delivery methods.

The aim of this pharmacokinetic research was to assess the effects of nanoencapsulation on the bioavailability and pharmacokinetic profile of orally administered LIRA in comparison to its subcutaneous administration in its free form. The study tracked plasma concentrations of LIRA over time following a single dose of either free LIRA (200 µg/kg) administered subcutaneously or Z-ERS-CS/LIRA Nps administered orally at an equivalent dose in rats. As depicted in Figure 4, plasma LIRA concentration-time profiles demonstrated a peak absorption at 4 h post-administration for both delivery methods, with the free LIRA achieving a peak plasma concentration of 109.49 ng/kg, and the Z-ERS-CS/LIRA reaching a slightly higher peak of 113.82 ng/kg (*p* > 0.05) (Table 3). The plasma concentration of LIRA from Z-ERS-CS/LIRA Nps displayed a more rapid decline phase than the free LIRA, attributable to the comparatively extended biodistribution phase of free LIRA from the subcutaneous tissue. Despite the inherent oral degradation challenges, including enzymatic breakdown and absorption kinetics within the gastrointestinal tract, the Nps formulation evidently safeguarded LIRA, enabling its effective transit to systemic circulation in a similar manner to subcutaneous administration. This is significant, as oral administration is generally less invasive and more favorably received by patients.

Key pharmacokinetic parameters, including C_max_, T_max_, T_1/2_ and AUC_0–24h_, are summarized in Table 3. The AUC_0–24h_ of free LIRA was only about 1.4-fold higher than that for the Z-ERS-CS/LIRA Nps (*p* < 0.05). Remarkably, the oral administration of LIRA, which would typically be completely degraded when loaded into Nps, was effectively absorbed, reaching excellent plasma levels, with a profile very similar to those observed in subcutaneous administration, especially in terms of C_max_. The Cl of the Z-ERS-CS/LIRA Nps was almost double that of the free LIRA, implying a more rapid elimination, explaining the AUC_0–24h_ results. The inclusion of chitosan (CS) in the Zein-ERS-Chitosan/LIRA nanoparticles not only may have facilitated mucoadhesion within the intestinal tract due to the polymer’s known bioadhesive properties but also the extensive surface area of the nanoparticles likely contributed to enhanced mucoadhesion, thus potentially improving the absorption profile. These findings underscore the potential of Z-ERS-CS/LIRA Nps to improve oral pharmacokinetic profiles, as also evidenced in the work of Shi et al. [48].

### 3.5. In Vivo Type 2 Diabetes Mellitus Experimental Model

The utilization of streptozotocin (STZ) to induce an experimental model of T2D presents inherent limitations when examining the hypoglycemic effects of LIRA [49]. This is because LIRA’s glucose-lowering action relies on the stimulation of insulin secretion by pancreatic β cells, which are extensively damaged by STZ. Nonetheless, this model has been widely adopted in various studies to evaluate LIRA’s therapeutic impact, as demonstrated by Eissa et al. (2023), Tong et al. (2021), Senduran et al. (2020), He et al. (2020), and Hendarto et al. (2012) [49,50,51,52,53].

**Figure 5:**
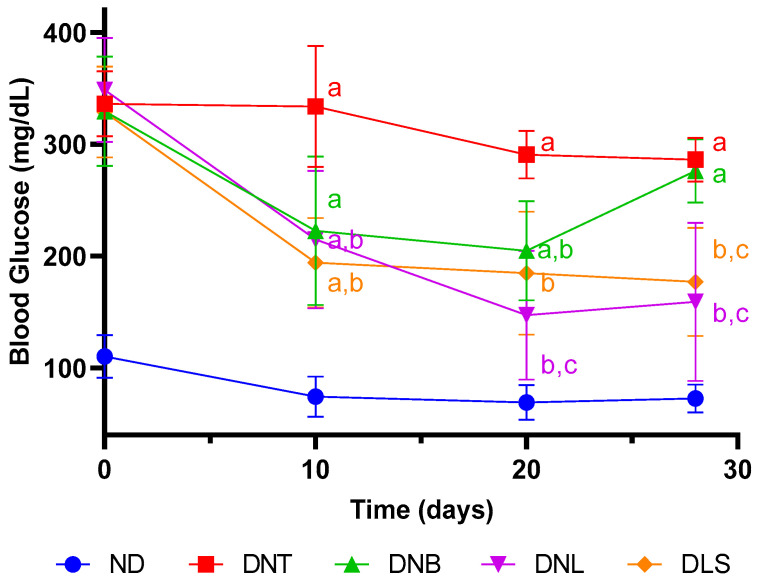
Plasma glucose levels over a 28-day treatment period. Groups: ND represents the non-diabetic control; DNT denotes the diabetic control without treatment; DNB illustrates the diabetic group receiving oral blank nanoparticles twice daily; DNL refers to the diabetic group receiving liraglutide-containing zein/eudragit-chitosan nanoparticles (Z-ERS-CS/LIRA Nps) (containing 72 µg of LIRA) orally twice daily; and DLS indicates the diabetic group treated with 72 µg of LIRA via subcutaneous injections twice daily. Values are expressed as mean ± standard deviation, with *n* = 10. Differences between groups were considered at *p* < 0.05. a, differences with the ND group; b, differences with the DNT group; c, differences with the DNB group.

The study’s outcomes distinctly accentuate the glycemic profile disparities among rat groups under different experimental paradigms, as depicted in Figure 5. The ND group, functioning as a control and receiving standard care, maintained normoglycemic states. Contrastingly, the DNT group, which did not receive any therapeutic intervention post-STZ induction, consistently exhibited blood glucose levels exceeding 300 mg/dL. This pronounced hyperglycemia solidifies the establishment of diabetes.

An examination of Figure 5 reveals variations in the DNB group glycemic trends over the course of the study. An initial decline in glucose concentrations was observed within the first 20 days, succeeded by a significant resurgence on day 30. This transient glycemic alleviation may be attributable to the presence of zein, which has been noted for its glucose-lowering properties in early stages post-administration, although this effect does not sustain over extended periods, aligning with the findings of previous research [54]. Zein’s high leucine content, an amino acid recognized for its acute insulinotropic effects, might explain this transient reduction in glucose levels [55,56].

The comparative analysis between the DNL and DLS-treated groups reveals notable glycemic patterns over time. The initial ten days showed a marked congruence in blood glucose reduction between the two groups. Yet, from day 20 onwards, a divergence emerged, with the DNL group displaying significantly lower glycemic levels compared to the DLS group (*p* < 0.05), indicating the oral treatment’s efficacy. The nanoformulation’s protective capacity seemingly facilitated drug absorption and subsequent systemic bioavailability, mirroring the effects of subcutaneous administration. This protection resulted in a notable reduction of approximately 25% in blood glucose levels, corroborating similar findings described in the work of Reboredo et al. [54].

In addition to the effects on glycemia, it is essential to address the influence of LIRA on body mass. Its contribution to reducing body adiposity and influencing the central appetite regulation mechanisms, resulting in weight reduction, opens new avenues for its use in obesity management alongside its glycemic regulatory functions [57]. These dual effects bolster LIRA’s comprehensive treatment profile for complex metabolic syndromes like diabetes and obesity.

**Figure 6:**
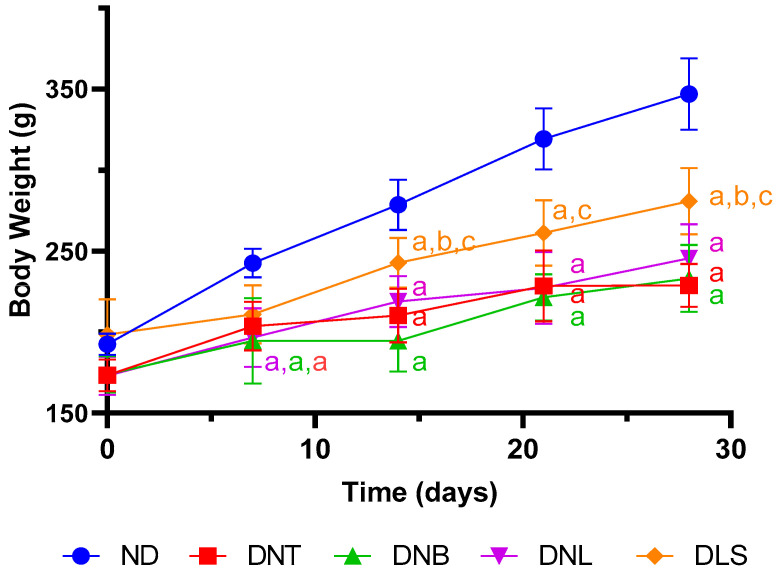
Animal body weight over a 28-day treatment period. Groups: ND represents the non-diabetic control; DNT denotes the diabetic control without treatment; DNB illustrates the diabetic group receiving oral blank nanoparticles (Nps) twice daily; DNL refers to the diabetic group receiving liraglutide-containing zein/eudragit-chitosan nanoparticles (Z-ERS-CS/LIRA) (containing 72 µg of LIRA) orally twice daily; and DLS indicates the diabetic group treated with 72 µg of LIRA via subcutaneous injections twice daily. Values are expressed as mean ± standard deviation, with *n* = 10. Differences between groups were considered at *p* < 0.05. a, differences with the ND group; b, differences with the DNT group; c, differences with the DNB group.

In terms of body mass, reflected in Figure 6, the ND group’s weight progression aligns with healthy growth expectations for rodents. In contrast, the DNT group showed weight stabilization, likely a consequence of metabolic dysfunctions stemming from insulin resistance, leading to compensatory metabolic processes like ketogenesis to sustain physiological homeostasis [58]. Regarding the DNB and DNL groups, no statistically significant disparities were identified. In contrast, the DLS group showed an increase in body weight when compared to the DNB group. The DNB group animals exhibited marginally lower weight gain compared to the DLS group. This could be tied to the initial decrease in blood glucose levels during the early treatment days without any concurrent diabetes medication. In STZ-induced diabetic animals that remain untreated, circulating insulin levels typically drop, leading to impairment of insulin-dependent anabolic tissue responses. Additionally, there tends to be a reduction in adipose tissue mass, which accelerates protein breakdown and curtails protein synthesis, collectively contributing to a diminished increase in skeletal muscle mass [59]. However, no significant weight differences were observed with the DNL group. This is corroborated by Song et al. (2023), who noted weight reduction in diabetic rats treated with oral LIRA nanocomposites [60] and Jakhar et al. (2023) [61], who observed similar effects with oral Nps containing LIRA. Senduran et al. (2020) also confirmed that LIRA encapsulated in PLGA Nps stabilized body weight in diabetic animals during treatment [51].

The results highlight the importance of considering different routes of administration when evaluating the therapeutic and physiological effects of pharmacological treatments [57,62]. Furthermore, these findings underscore the significant role of oral Z-ERS-CS/LIRA Nps in eliciting comparable effects on blood glucose levels and body weight in diabetic rats, compared to subcutaneous LIRA administration.

## 4. Conclusions

The study successfully formulated zein nanoparticles using Eudragit RS100 and chitosan as stabilizers for encapsulating liraglutide (Z-ERS-CS/LIRA). These nanoparticles exhibited an average diameter suitable for oral administration (<350 nm), with high size uniformity and a highly positive zeta potential, suggesting nanoparticle stability. The encapsulation efficiency was around 41%, aligning with the literature on hydrophilic molecule encapsulation. In vitro release tests demonstrated the nanoparticles’ significant resistance and stability in digestive enzyme environments. Pharmacokinetic tests revealed that the oral administration of Z-ERS-CS/LIRA nanoparticles resulted in a pharmacokinetic profile comparable to liraglutide subcutaneous application. Moreover, in vivo tests on a type 2 diabetes model showed Z-ERS-CS/LIRA substantial efficacy in reducing blood glucose levels compared to subcutaneous liraglutide treatments. These findings indicate that the nanoparticles developed could be a highly promising system for the oral delivery of liraglutide for the treatment of type 2 diabetes mellitus.

## Figures and Tables

**Table 1:** Physicochemical properties of zein/eudragit-chitosan nanoparticles: comparison between blank nanoparticles (Z-ERS-CS) and liraglutide-containing nanoparticles (Z-ERS-CS/LIRA) (*n* = 6).

Sample	Mean Size ± SD (nm)	PDI ± SD	Zeta Potential ± SD (mV)	EE% ± SD
Z-ERS-CS	225.9 ± 11.7 ^a^	0.070 ± 0.015 ^a^	+40.5 ± 2.6 ^a^	-
Z-ERS-CS/LIRA	238.6 ± 10.2 ^a^	0.099 ± 0.026 ^a^	+40.9 ± 3.6 ^a^	41.1 ± 2.6

^a^ Same letters mean statistical equality, analyzed by column (ANOVA and post-Tukey test and *p* < 0.05). EE = Encapsulation efficiency; PDI = polydispersity index; SD = standard deviation.

**Table 2:** Comparative analysis of mathematical models for in vitro liraglutide release dynamics.

Model	a	b	r	n
Zero order	1.82	24.1	0.780	
First order	0.05	3.12	0.588	
Second order	−0.002	0.048	0.392	
Third order	−0.0001	0.003	0.261	
Korsmeyer-Peppas	0.394	2.991	0.935	0.394
Higuchi	0.500	2.853	0.593	
Weibull	0.487	−1.497	0.961	
Hickson-Crowell	0.055	2.856	0.656	

a = Slope coefficient; b: Intercept coefficient; r: Correlation coefficient; n: Release exponent.

**Table 3:** Comparative plasma concentration-time profiles following a single dose administration of free liraglutide (LIRA) subcutaneously versus LIRA-containing zein/eudragit-chitosan nanoparticles (Z-ERS-CS/LIRA) orally in rats (*n* = 6). Both formulations were administered at a dose of 200 µg/kg of LIRA.

Pharmacokinetic Parameters	Z-ERS-CS/LIRA Nps	Free LIRA
C_max_ (ng/mL)	113.82 ± 25.24 ^a^	109.49 ± 27.54 ^a^
T_max_ (h)	4.0 ^a^	4.0 ^a^
K_el_ (1/h)	0.134 ± 0.039 ^a^	0.115 ± 0.019 ^a^
Cl (L/h)	0.284 ± 0.077 ^a^	0.183 ± 0.015 ^b^
T_1/2_ (h)	5.50 ± 1.38 ^a^	6.09 ± 0.96 ^a^
AUC_0–24h_ (ng.h/mL)	692.86 ± 37.59 ^a^	977.79 ± 46.23 ^b^

C_max_: Maximum concentration; T_max_: Time to reach maximum concentration; AUC_0–24h_: Area under the plasma concentration-time curve; T_1/2_: Half-life; Kel: Elimination rate constant Cl: Clearance. ^a,b^ Same letters mean statistical equality and different letters statistical inequality, analyzed by line (ANOVA and post-Tukey test and *p* < 0.05).

## Data Availability

Data will be available on request.
